# Valorization of ligustrum species: biosynthesis, metabolic engineering, and pharmacology of bioactive compounds

**DOI:** 10.1186/s40643-026-01042-3

**Published:** 2026-03-27

**Authors:** Dipeng Li, Chenyang Lu, Yan Zhang, Yunfei Yu, Chenghao Fei, Peng Chen, Rao Fu, Mao Wu, Peina Zhou

**Affiliations:** 1 Wuxi Affiliated Hospital of Nanjing University of Chinese Medicine, Wuxi, 214071 China; 2 Hangzhou Ninth Hospital, Hangzhou, 310000 China; 3 Nanjing Research Institute for Comprehensive Utilization of Wild Plants, All China Federation of Supply and Marketing Cooperatives, Nanjing, 210042 China; 4 College of Horticulture, Nanjing Agricultural University, Nanjing, 210095 China

**Keywords:** *Ligustrum*, Bioactive components, Biosynthesis, Regulation, Metabolic engineering, Pharmacological activity

## Abstract

The genus *Ligustrum* (Oleaceae) encompasses woody plants with both medicinal and edible uses, distinguished by a wide range of bioactive compounds, including triterpenoids, phenylethanoid glycosides, flavonoids, and other active constituents. These metabolites demonstrate multi-target pharmacological effects, such as anti-inflammatory, antioxidant, antitumor, and anti-osteoporotic activities. In traditional medicine, species like *L. lucidum* and *L. robustum* are well-documented for their therapeutic roles in nourishing the liver and kidneys, enhancing vision, darkening hair, and serving as functional tea ingredients. Beyond their medicinal and health-promoting properties, *Ligustrum* species are also employed as ornamental plants, bioindicators of atmospheric pollution, algicidal agents, and feed additives. Given the increasing global demand and underutilization of *Ligustrum* resources, there is an urgent need to establish a sustainable supply framework focused on alternative strategies such as metabolic engineering, synthetic biology, and environmentally friendly manufacturing. This review encapsulates recent progress in the exploration of chemical diversity, pharmacological characteristics, omics-based analyses, and biosynthetic research pertaining to *Ligustrum*. It proposes an integrated methodology that amalgamates multi-omics approaches, synthetic biology, and environmentally sustainable manufacturing processes to advance strategies for whole-plant valorization and germplasm conservation. The objective is to establish a theoretical framework and technical paradigm to facilitate the comprehensive exploitation and sustainable utilization of *Ligustrum* as a medicinal resource.

## Introduction

The genus *Ligustrum* (Oleaceae) encompasses a range of evergreen and deciduous shrubs to small trees, which are esteemed for their ornamental, medicinal, and ecological importance, constituting a significant element of the temperate-subtropical flora. The primary center of its distribution is located in East Asia, with the greatest species diversity found in the Sino-Himalayan region, extending towards Europe and North Africa (Gao et al. 2013; Tan 2020). Within this genus, several species are recognized for their medicinal properties, including *L. lucidum* Ait., *L. robustum* (Roxb.) Blume, *L. japonicum* Thunb., and *L. purpurascens* Y.C. Yang (Gao et al. 2013; Kim et al. 2022; Qiao et al. 2019; Song et al. 2012). Notably, the dried ripe fruits of *L. lucidum*, referred to as “Nüzhenzi” in traditional Chinese medicine (TCM), are known for their hepatorenal-tonifying, vision-enhancing, and hair-darkening effects. Additionally, the leaves of *L. robustum* are processed locally into “Kudingcha”, a functional tea acclaimed for its health-promoting and dietary benefits, often dubbed “longevity tea” and “beauty tea,” highlighting its dual medicinal and edible properties (Ling et al. 2022, 2023). As a result, *Ligustrum* has emerged as a highly promising natural resource for the pharmaceutical, functional food, and cosmetic industries. Products derived from *Ligustrum* species, especially those containing salidroside and triterpenoids such as oleanolic acid and ursolic acid, hold considerable commercial interest in the global nutraceutical, cosmetic, and pharmaceutical markets, which were driven by demand for natural and sustainable bioactive ingredients.

As of now, more than 200 chemical compounds have been identified in *Ligustrum* plants, spanning structural classes such as triterpenoids, phenylethanoid glycosides, iridoids, flavonoids, and volatile oils. These compounds collectively form the material basis for the plants’ multi-target pharmacological activities, which include anti-inflammatory, antimicrobial, antiviral, antioxidant, antitumor, anti-osteoporotic, and immunomodulatory effects (Chen et al. 2024a, b; Wang et al. 2022; Zuo et al. 2025). Among these compounds, triterpenoids (e.g., oleanane- and ursane-type) are significant contributors to antioxidant, hypoglycemic, and anti-osteoporotic activities (Cao et al. 2018). Phenylethanoid glycosides (e.g., salidroside and verbascoside) primarily facilitate lipid-lowering, antiviral, and neuroprotective effects (Song et al. 2012), while flavonoids (e.g., luteolin and quercetin derivatives) demonstrate notable efficacy in antioxidant and anti-inflammatory/analgesic functions (Kang et al. 2021). The 2025 edition of the *Chinese Pharmacopoeia* identifies specnuezhenide as the marker compound for Ligustri Lucidi Fructus (LLF, 女贞子) and salidroside for its processed slices. Nevertheless, non-traditional medicinal components, such as leaves and stems, which often contain comparable or even greater concentrations of bioactive constituents, remain underutilized. This underscores a significant untapped potential for resource development (Chinese Pharmacopoeia and Commission 2025).

In the current omics era, significant progress has been made in elucidating the genomic, complete chloroplast genome, transcriptomic, and metabolomic profiles of *Ligustrum* species, thereby establishing a comprehensive omics framework for deciphering the biosynthetic pathways of their key pharmacologically active constituents (Jin et al. 2022; Li et al. 2025; Wang et al. 2018; Zhou et al. 2024). Nevertheless, contemporary research predominantly emphasizes phytochemical characterization and pharmacological validation. Systematic investigations into the enzymatic mechanisms, regulatory networks, and synthetic biology-based engineering of core metabolic fluxes—particularly those involved in the biosynthesis of triterpenoid saponins, iridoids, and phenylethanoid glycosides—remain insufficient. These knowledge gaps represent critical bottlenecks that impede both the sustainable utilization of resources and the heterologous biosynthesis of bioactive compounds. Considering these factors, it is essential to incorporate multi-omics and synthetic biology methodologies to elucidate the functional roles of pivotal enzymatic genes, reconstruct metabolic pathways, and explore value-added utilization models for non-fruit tissues such as leaves and stems. This integrated approach would enable a comprehensive development of *Ligustrum* resources by promoting whole-plant utilization, full-spectrum component exploitation, and optimization of the entire value chain.

This review systematically consolidates contemporary understanding of the chemical structural diversity of key bioactive constituents in *Ligustrum* species, alongside their pharmacological profiles, recent advancements in omics-based characterization, and the current status of biosynthetic and metabolic engineering research. Additionally, it provides forward-looking perspectives on the conservation of germplasm resources, eco-friendly production of active compounds, and potential industrialization pathways. The synthesis seeks to establish both theoretical foundations and technical paradigms for the comprehensive exploration and sustainable utilization of medicinal plants within this genus.

### Classification of ***Ligustrum*genus**

The genus *Ligustrum* (Oleaceae) encompasses approximately 50 species distributed throughout Eurasia, with its primary center of diversity located in the warm temperate regions of Asia. In China, this genus is particularly widespread, comprising 38 species, of which 14 have been recognized for their medicinal significance: *L. lucidum* Ait., *L. robustum* (Roxb.) Blume, *L. quihoui* Carr., *L. pedunculare* Rehd., *L. purpurascens* Y.C.Yang, *L. japonicum* Thunb., *L. pricei* Hayata, *L. henryi* Hemsl., *L. delavayanum* Hariot, *L. ovalifolium* Hassk., *L. vulgare* L., *L. vicaryi* Hort., *L. sinense* Lour., and *L. obtusifolium* Sieb (Tan et al. 2020). Among these species, *L. lucidum* has been the subject of extensive research. Its dried fruits, referred to as “Nüzhenzi” were initially documented in the Shennong’s Classic of Materia Medica (《神农本草经》) and later in the Compendium of Materia Medica (《本草纲目》). This superior-grade medicinal material is reputed for its ability to “tonify the middle, calm the five viscera, nourish essence, and eliminate various diseases.” It has been consistently documented in historical Chinese materia medica and all editions of the *Chinese Pharmacopoeia* from 1963 to 2025 (Cao et al. 2023; Chinese Pharmacopoeia and Commission 2025; Gao et al. 2015). Kudingcha, a term encompassing traditional herbal teas that are second only to Camellia tea in popularity within China and commonly referred to as Kuding Tea or Bitter Nail Tea, has been utilized both as a beverage and as ethnomedicine in the southwestern and southern regions of China for over 2,000 years. This practice exemplifies the concept of medicinal-food homology (Ling et al. 2022). Within the genus *Ligustrum*, the leaves of *L. pedunculare*, *L. japonicum*, *L. robustum*, and *L. purpurascens* are identified as botanical sources of Kudingcha, with *L. robustum* being the primary source of dried leaves for this functional tea (He et al. 2017; Lu et al. 2022).

### The main bioactive components and distribution of ***Ligustrum*** genus

**Fig. 1:**
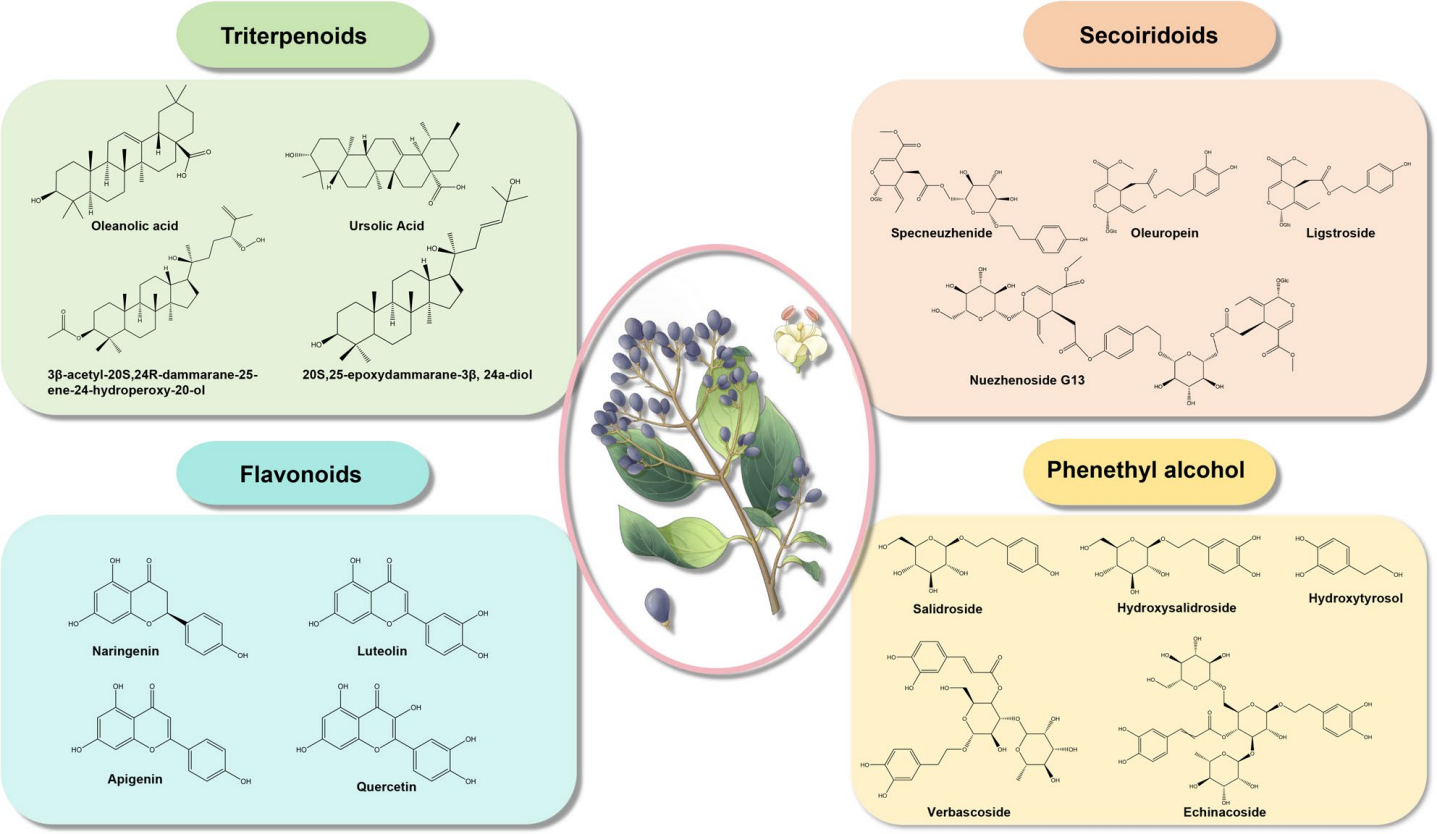
The main bioactive components of *Ligustrum* genus

The genus *Ligustrum* has been documented to contain over 200 chemical compounds, predominantly consisting of phenylethanoid derivatives in both ester and glycosidic forms, iridoids, triterpenoids, flavonoids, volatile oils, and polysaccharides (Fig. 1). The iridoids present in *Ligustrum*, which are primarily water-soluble, belong to the secoiridoid class, with 51 compounds identified that share similar fundamental structures and biological activities (Cao et al. 2023; Liu and Zou 2022). Notable secoiridoids include oleuropein, specnuezhenide, and nuezhenide G13, with specnuezhenide serving as the representative marker compound for the quality assessment of LLF in the *Chinese Pharmacopoeia*, which mandates a minimum content of 0.70% (Cao et al. 2023). However, most studies focus on a few marker compounds, leaving the bioactivity profiles of many structurally similar but less abundant iridoids largely unexplored. Triterpenoids present in LLF can be structurally categorized into tetracyclic and pentacyclic types, with a total of 42 compounds currently identified. The pentacyclic triterpenoids encompass derivatives of oleanane, ursane, and lupane, while the tetracyclic compounds are predominantly represented by dammarane-type variants. Pharmacological investigations indicate that pentacyclic triterpenoids exhibit more extensive and significant bioactivities in comparison to their tetracyclic counterparts. Notably, oleanolic acid and ursolic acid, which are found in relatively high concentrations within LLF, have been recognized as the principal bioactive constituents (Du et al. 2024; Ji et al. 2021). A critical gap exists in systematically comparing the bioactivities of diverse tetracyclic versus pentacyclic triterpenoids within the *Ligustrum* context. The synergistic or antagonistic interactions between different triterpenoid classes are also virtually unstudied.

The phenylethanoid derivatives found in *Ligustrum* are predominantly represented by phenylethanoid glycosides, with 16 compounds of this class currently documented. Salidroside is the characteristic constituent of this group, characterized by a glucosyl core with ester and O-glycosidic linkages, typically substituted with hydroxyl or methoxyl groups on the phenethyl moiety and frequently esterified with cinnamoyl groups. In addition to salidroside, other quantitatively significant compounds include hydroxysalidroside, tyrosol, hydroxytyrosol, verbascoside, echinacoside, and ligupurpuroside A and B (Chen et al. 2017; Zhou et al. 2024). Notably, in *L. robustum*, verbascoside, ligupurpuroside A, and B collectively constitute up to 10% of the leaf dry weight (Li et al. 2012). This exceptionally high yield in *L. robustum* highlights significant interspecies variation. So, can the *L. robustum* serve as a reliable source for the industrial extraction of these compounds? Moreover, the ecological or physiological mechanisms underlying tissue-specific accumulation remain an unexplored area of research. Flavonoids represent another significant class of bioactive constituents in *Ligustrum*, with 15 distinct compounds isolated from various plant parts, including naringenin, luteolin, apigenin, quercetin, rutin, and apigenin-7-O-glucoside. Of particular interest is ligustroflavone, a structurally unique flavonoid considered taxonomically characteristic of the *Ligustrum* genus (Bi et al. 2023). Pharmacological research has elucidated its substantial anti-inflammatory, anti-fibrotic, and antioxidant properties, highlighting its potential for therapeutic application (Bi et al. 2023; Kang et al. 2021). Despite its taxonomic significance, the distribution pattern of ligustroflavone across the entire genus is not well-mapped.

The quantitative assessment of specific bioactive compounds, including the iridoid specnuezhenide, the phenylethanoid glycoside salidroside, and the triterpenoids oleanolic acid and ursolic acid, has been established as a comprehensive quality control measure for medicinal materials derived from *Ligustrum*. Research indicates that alcohol processing of LLF does not significantly affect the levels of lipophilic triterpenoids, such as ursolic acid and oleanolic acid. However, during a 24-hour alcohol-steaming process, iridoid compounds such as specnuezhenide, nuezhenide, and oleuropein tend to decrease due to hydrolysis, while the concentrations of most phenylethanoids, including salidroside, tend to increase (Xue et al. 2024).

In addition to alcohol processing, alternative preparation methods utilizing salt and vinegar have been explored. A comparative analysis of oleanolic acid content across these methods reveals the following hierarchy: alcohol-processed > salt-processed > vinegar-processed > unprocessed LLF (Chen and Weng 2021). The extent of steaming further affects triterpenoid yields, with quantitative studies indicating the sequence: alcohol-steamed > lightly steamed > vinegar-steamed > raw LLF. Processed samples consistently exhibit higher oleanolic acid content compared to unprocessed LLF. While these comparative studies exist, a major research gap is the lack of standardized, multi-factorial studies that dissect the individual and interactive effects of processing parameters on a broader spectrum of metabolites beyond a few markers. Studies on tissue-specific distribution reveal differential accumulation patterns, with whole fruits, exocarps, and kernels containing 33.1%, 13.5%, and 48.7% of total triterpenoids (oleanolic acid and ursolic acid), respectively. Notably, the exocarp demonstrates superior extraction efficiency, yielding 24.34 ± 2.09 mg/g of oleanolic acid and 7.82 ± 0.09 mg/g of ursolic acid, which is approximately four times higher than the whole-fruit extracts for both compounds (Dong et al. 2022). A comparative phytochemical analysis of *L. lucidum* fruits, leaves, and stems demonstrates distinct patterns of tissue-specific compound accumulation. The fruits are characterized by significantly elevated levels of loganate, secologanoside, nuzhenal C, luteolin, iso-oleonuezhenide, and dammarenediol-II relative to the leaves and stems. Additionally, triterpenoids such as oleanolic acid and β-amyrin preferentially accumulate in the fruits and stems, with notably reduced concentrations observed in the leaves (Zuo et al. 2025). Analyses of the developmental stages of *L. lucidum* fruits reveal dynamic biosynthetic patterns of key active constituents. The iridoids, specnuezhenide and nuezhenide G13, alongside phenylethanoids such as tyrosol and salidroside, exhibit distinct accumulation trajectories throughout fruit maturation. Post-harvest processing exerts a significant impact on metabolite stability: phenylethanoid glycosides experience substantial degradation during drying, whereas iridoid compounds demonstrate increased concentration in dried materials (Zhou et al. 2024). The dynamic changes during development and post-harvest are critically understudied from a mechanistic perspective. The key enzymes and genes driving these shifts in metabolite profiles are unknown. This gap hinders the development of strategies to optimize harvest timing and post-harvest handling to maximize the yield of desired compounds. The content of bioactive constituents in *L. lucidum* fruits varies significantly based on agroclimatic conditions, harvest timing, post-harvest processing techniques, and the specific plant tissues collected. Therefore, there is an urgent need for controlled, systematic research to identify and quantify the impact of individual environmental factors such as light, temperature, and soil nutrients on the metabolomics of *Ligustrum* species.

## Biosynthesis and regulation of the main bioactive constituents of Ligustrum

### Biosynthesis of secoiridoids and triterpenoids

**Fig. 2:**
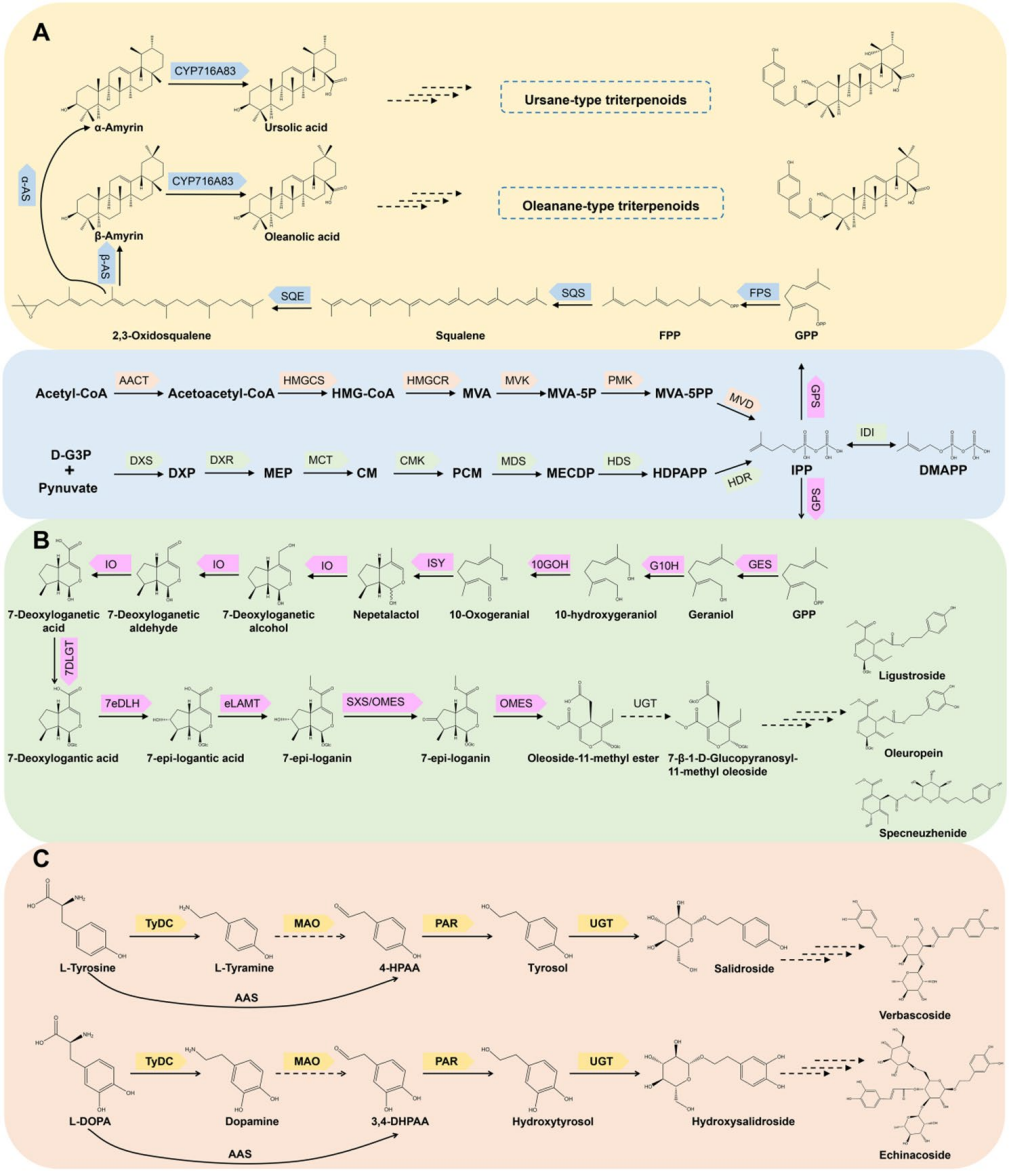
Biosynthetic pathways of main bioactive constituents of *Ligustrum*. (**A**) Biosynthetic pathways of ursane-type and oleanane-type triterpenoids. (**B**) Biosynthetic pathways of secoiridoids. (**c**) Biosynthetic pathways of phenylethanoid glycosides

In *Ligustrum* species, the biosynthesis of terpenoid compounds is initiated through two precursor pathways: the mevalonate (MVA) and the methylerythritol phosphate (MEP) pathways. These pathways converge to generate the universal isoprenoid precursors, isopentenyl pyrophosphate (IPP) and its isomer, dimethylallyl pyrophosphate (DMAPP), via a series of enzymatic reactions (Chen et al. 2024a, b). Subsequently, under the catalysis of geranyl diphosphate synthase (GPPS), IPP is transformed into geranyl pyrophosphate (GPP), which acts as a central intermediate for the synthesis of various terpenoid derivatives. Within the secoiridoid biosynthetic pathway, geranyl diphosphate (GPP) undergoes dephosphorylation to generate geraniol. This compound is subsequently modified through a series of enzymatic reactions involving geraniol 10-hydroxylase (G10H), 10-hydroxygeraniol oxidoreductase (10GHO), and iridoid synthase (ISY), ultimately resulting in the formation of nepetalactol. Nepetalactol serves as an intermediate that is further transformed by iridoid oxidase (IO) and 7-deoxyloganetic acid glucosyltransferase (7DLGT) to produce 7-deoxyloganic acid. This compound undergoes additional enzymatic modifications through the action of 7-deoxy-loganic acid 7-epihydroxylase (7eDLH), 7-epi-loganic acid methyltransferase (eLAMT), oleoside methyl ester synthase (OMES), and secoxyloganin synthase (SXS), leading to the synthesis of oleoside-11-methyl ester (OME). Ultimately, glycosylation and esterification processes convert OME into the characteristic secoiridoids, such as oleuropein and ligstroside (Table 1, Fig. 2) (Li et al. 2025; Zhou et al. 2024).

In the biosynthesis of triterpenoids, geranyl pyrophosphate (GPP) undergoes elongation by farnesyl pyrophosphate synthase (FPPS) to yield farnesyl pyrophosphate (FPP). Subsequently, FPP is transformed by the sequential action of squalene synthase (SQS) and squalene epoxidase (SQE) into the pivotal intermediate, 2,3-oxidosqualene. This intermediate acts as a crucial branching point for the synthesis of pentacyclic triterpenoids. Specifically, β-amyrin synthase, in conjunction with CYP716A83, facilitates the production of oleanolic acid, whereas α-amyrin synthase and CYP716A83 are responsible for the formation of ursolic acid. Both oleanolic and ursolic acids can undergo further modifications to yield a diverse array of derivatives (Fig. 2) (Chen et al. 2024a, b; Liu et al. 2020).

Although the upstream and certain downstream pathways involved in the biosynthesis of secoiridoids and triterpenoids in *Ligustrum* have been elucidated, substantial knowledge gaps persist concerning the modifications occurring in the later stages. In contrast to the well-studied early steps such as the MVA/MEP pathways and initial cyclization reactions, research on the subsequent modification and regulatory steps lags significantly. Critical unresolved processes include the precise glycosylation and acylation mechanisms responsible for the conversion of OME into oleuropein or ligstroside. Current understanding largely relies on homology-based predictions or indirect evidence, lacking functional validation of the relevant glycosyltransferases and acyltransferases within *Ligustrum* systems. Moreover, the substrate specificity, enzyme kinetics, and subcellular localization of these late modification steps remain unknown. Furthermore, compared with the regulatory studies of analogous pathways in other medicinal plants, the regulatory networks that control the biosynthesis of these specialized metabolites in *Ligustrum* have yet to be investigated. A systematic examination of these underexplored biosynthetic and regulatory elements will lay the groundwork for metabolic engineering and the sustainable production of these valuable phytochemicals. Addressing these gaps will not only deepen our understanding of the diversity of secondary metabolism in *Ligustrum* but also facilitate the heterologous production of target compounds through synthetic biology strategies.

**Table 1:** The key catalytic enzymes of pathways in Fig. 2

Pathway	Background color	Key catalytic enzyme
Upstream terpenoid pathway – MVA pathway	Light blue	AACT, HMGCS, HMGCR, MVK, PMK, MVD
Upstream terpenoid pathway – MEP pathway	Light blue	DXS, DXR, MCT, CMK, MDS, HDS, HDR, IDI
Secoiridoid biosynthetic pathway	Light green	GPPS, GES, G10H, 10GOH, ISY, IO, 7DLGT, 7eDLH, eLAMT, SXS, OMES, UGT
Pentacyclic triterpenoid biosynthetic pathway	Light yellow	FPS, SQS, SQE, α-AS, β-AS, CYP716A83
Phenylethanoid glycoside biosynthetic pathway	Apricot	TyDC, MAO, PAR, AAS, UGT

### Biosynthesis of phenethyl alcohol

Phenethyl alcohol and its glycosides are among the primary bioactive components found in *Ligustrum* species. These compounds are characterized by a hydroxyphenylethyl moiety linked to a glucopyranose unit via a β-glycosidic bond, which may be further modified with additional sugar residues (Fu et al. 2008). The core structure is often altered by hydroxycinnamic acids and other phenolic acid derivatives (Agar and Cankaya 2020). Salidroside is the prototypical compound of this class, with structurally related derivatives such as hydroxysalidroside, verbascoside, echinacoside, and ligupurpuroside A and B also present in significant quantities (Chen et al. 2024a, b; Wu et al. 2020). This profile is distinct from other medicinal plants rich in phenylethanoid glycosides, such as *Cistanche*, and *Rehmannia*, highlighting genus-specific diversification patterns. The biosynthesis of these compounds originates from tyrosine, where L-tyrosine undergoes decarboxylation by tyrosine/dopa decarboxylase (TyDC) to form tyramine. This is followed by oxidation via monoamine oxidase (MAO) to produce 4-hydroxyphenylacetaldehyde (4-HPAA), which is subsequently reduced by phenylacetaldehyde reductase (PAR) to yield tyrosol. Glycosylation at the C8 position, facilitated by UDP-glucose 8-O-glucosyltransferase (T8GT), results in the formation of salidroside (Torrens-Spence et al. 2018). The biosynthesis of hydroxysalidroside proceeds via a parallel pathway, beginning with L-3,4-dihydroxyphenylalanine through a series of reactions catalyzed by a consistent enzyme system (TyDC, MAO, PAR, and T8GT) (Torrens-Spence et al. 2018; Zhou et al. 2024). Subsequent structural diversification is achieved through a sequence of post-glycosylation modifications: initially, salidroside undergoes acylation to form osmanthuside A, which is then glycosylated by UGT79G7 to yield osmanthuside B. Further glycosylation by UGT79A19 results in the formation of ligupurpuroside B. Osmanthuside B and ligupurpuroside B are subsequently subjected to dihydroxylation, producing verbascoside and ligupurpuroside A, respectively (Yang et al. 2021). Recent studies propose aromatic aldehyde synthase (AAS) as an alternative enzyme capable of directly converting L-tyrosine and L-DOPA into their corresponding aldehydes (4-HPAA and 3,4-DHPAA), potentially streamlining the pathway. However, the functional delineation between TyDC and AAS remains problematic due to high sequence homology within the aromatic amino acid decarboxylase (AAAD) family, underscoring a critical need for precise biochemical characterization beyond in silico predictions (Torrens-Spence et al. 2018). While several downstream glycosyltransferases, such as T8GT, UGT79G7, and UGT79A19, in *Ligustrum* have been functionally characterized, key upstream enzymes, including TyDC, MAO), PAR, and AAS, remain unverified (Fig. 2). Furthermore, the regulatory logic governing the branching flux from salidroside towards diverse end-products (e.g., verbascoside vs. ligupurpurosides) is completely unknown. It remains unclear whether this divergence is controlled by substrate specificity of the modifying enzymes, compartmentalization, or transcriptional regulation of specific pathway branches. This knowledge gap impedes a systems-level understanding and highlights the necessity for a comprehensive elucidation of both the early and late biosynthetic steps, including their regulation, to enable metabolic engineering efforts for sustainable production of these valuable compounds.

### Metabolic engineering of the main bioactive compounds in ***Ligustrum***

Although *Ligustrum* species are known for producing a wide array of bioactive compounds, traditional extraction methods are often labor-intensive and unsustainable. Similarly, chemical synthesis is hindered by complex processes, high energy consumption, and low yields. However, advancements in metabolic engineering have facilitated the development of plant and microbial cell factories, offering promising alternatives for the sustainable production of these phytochemicals. This innovative approach not only addresses resource limitations but also enables efficient, environmentally friendly, controllable, and scalable manufacturing of natural products. Recent developments in microbial metabolic engineering have introduced a variety of strategies for the heterologous biosynthesis of these valuable bioactive compounds (Table **2**), marking a significant paradigm shift in the production of natural products.

### Metabolic engineering of phenethyl alcohol compounds

Salidroside, a prominent phenethyl alcohol compound found in *Ligustrum* species, has attracted considerable scholarly interest due to its diverse pharmacological properties, such as antioxidant, anti-fatigue, anti-aging, anticancer, anti-inflammatory, and neuroprotective effects, rendering it valuable for applications in the cosmetic, nutraceutical, and pharmaceutical industries (Chen et al. 2020; Lai et al. 2024; Liu et al. 2023; Pan et al. 2022; Xiong et al. 2021). In response to growing market demand and the elucidation of critical enzymatic steps within its biosynthetic pathway, synthetic biology approaches utilizing microbial cell factories have emerged as a promising solution for the efficient production of salidroside (Zeng et al. 2024). This innovative strategy presents distinct advantages, including reduced production cycles, enhanced process control, and scalable manufacturing capabilities, thereby addressing existing supply constraints.

To date, the de novo biosynthesis of salidroside has been successfully accomplished in various microbial hosts, including *Escherichia coli* and *Saccharomyces cerevisiae* and plant host *Nicotiana benthamiana*. Initial endeavors focused on the introduction of key enzymes—phenylpyruvate decarboxylase ARO10 from *S. cerevisiae* and glucosyltransferase UGT73B6 from *Rhodiola crenulata*—into *E. coli*, resulting in the production of 56.9 mg/L salidroside from glucose through optimized fermentation processes (Bai et al. 2014). A syntrophic *E. coli* co-culture system, consisting of aglycon (AG)- and glycoside (GD)-producing strains engineered with tyrosol and salidroside biosynthetic pathways, respectively, achieved a production level of 6.03 g/L salidroside after 129 h (Liu et al. 2018). Transient co-expression of *Rhodiola rosea* 4-hydroxyphenylacetaldehyde synthase (4-HPAAS) and T8GT in *N. benthamiana* yielded salidroside at 2% of dry weight (20 mg/g DW) in the leaves. In contrast, co-expression of codon-optimized 4-HPAAS and T8GT in *S. cerevisiae* produced salidroside at a titer of 1.5 mg/L (Torrens-Spence et al. 2018). Significant improvements in yield were realized through genomic integration strategies: Sisi Liu et al. utilized multi-copy integration of UGT85A1, which increased production from 120 mg/L to 2.42 g/L, with further optimization reaching 9.34 g/L after 120 h via integration into non-coding regions (Liu et al. 2022). Huayi Liu and colleagues developed a modularly engineered *Saccharomyces cerevisiae* strain expressing *RrU8GT33*, achieving the highest reported titer of 26.55 g/L at 168 h (Liu et al. 2021). To address challenges related to industrial scalability, Jingwen Zhou and collaborators engineered *E. coli* by incorporating ARO10 and ADH6 for de novo synthesis of tyrosol (3.3 g/L). Subsequently, they introduced the glucosyltransferase AtUGT85A1 and overexpressed phosphoglucomutase and UDP-glucose pyrophosphorylase (galU) to facilitate salidroside biosynthesis. Through semi-rational mutagenesis (A21G) of AtUGT85A1, production was further enhanced to 16.8 g/L (Zeng et al. 2024). These microbial fermentation and plant platforms signify a paradigm shift from conventional plant extraction and chemical synthesis methods, offering superior efficiency and environmental sustainability that align with principles of green manufacturing. Moreover, this research establishes a foundational framework for the microbial production of other valuable natural products.

### Metabolic engineering of terpenoids compounds

Traditional methods for terpenoid production, which rely on plant extraction or chemical synthesis, encounter significant challenges, such as intricate processes, low efficiency, and limited scalability. These limitations underscore the need for alternative synthetic methodologies. Recent advancements in synthetic biology have introduced innovative solutions for the efficient production of terpenoids. Among these, heterologous reconstruction strategies have gained prominence in triterpenoid biosynthesis. Plant transient expression systems, particularly those utilizing *N. benthamiana*, have emerged as valuable platforms due to their well-characterized genetic profiles and operational simplicity. These systems have been successfully employed to reconstruct pathways for a variety of bioactive terpenoids, including monoterpenes, sesquiterpenes, and diterpenes (McClune et al., 2025; Xu et al. 2024; Zhang et al. 2023). Notably, a research team from Stanford successfully integrated 17 genes into tobacco plants to produce the taxol precursor baccatin III at yields of 30 µg/g, which are comparable to the natural levels found in yew trees(McClune et al., 2025). Furthermore, a range of bioactive compounds, such as liquiritin apioside (5.47 mg/g DW) and iso-liquiritin apioside (4.73 mg/g DW) from *Glycyrrhiza uralensis*, crocin (3.5 mg/g DW) and picrocrocin (8 mg/g DW) from saffron, and the saponin adjuvant QS-7 (7.9 µg/g DW), have been synthesized using tobacco-based systems (Marti et al. 2020; Reed et al. 2023; Wang et al. 2023). Presently, research in metabolic engineering concerning pentacyclic triterpenoids in *Ligustrum* is sparse, with only isolated studies addressing the heterologous production of aglycone precursors like oleanolic acid and ursolic acid. After transient co-expression of AtHMGR1cd-S577A in tobacco using the Tsukuba system, the content of triterpenoids increased, resulting in the production of oleanolic acid at a level of 30.8 mg/g DW (Romsuk et al. 2022). When MtCYP716A12 was co-expressed with MtCPR1 and MtCPR2 in tobacco, the yields of ursolic acid were 35.87 ± 2.32 mg/g DW and 34.35 ± 2.35 mg/g DW, respectively (Romsuk et al. 2024).

Microbial cell factories, notably *S. cerevisiae*, *Yarrowia lipolytica*, and *E. coli*, have emerged as leading platforms for duction of triterpenoids. This predominance is attributed to their advanced genetic manipulation tools, rapid growth rates, and scalability in cultivation processes. These microbial systems demonstrate exceptional proficiency in expressing genes derived from plants and synthesizing plant metabolites(Bureau et al. 2023; Mai et al. 2025; Ming et al. 2025) For example, researchers were able to optimize the biosynthetic pathway, resulting in a *Yarrowia lipolytica* cell factory that produced high levels of β-elemene (5.08 g/L), (S)-linalool (10.9 g/L) produced in *Pantoea ananatis*, dammarenediol-II (15 g/L) produced in eukaryotic yeasts, ginsenoside Rh2 with 2.3 g/L in 10 L fed-batch fermentation and so on (Li et al. 2023, 2024; Shi et al. 2021). Oleanolic acid and ursolic acid can be produced in considerable quantities within microbial systems. The researchers first introduced CrAS, CrAO, and AtCPR1 into yeast. They then increased the levels of cytoplasmic acetyl-CoA, fine-tuned the copy numbers of ERG1 and CrAS, targeted these key enzymes to lipid droplets, and enhanced the regeneration of NADPH. As a result, they were able to obtain 1.13 g/L of UA and 0.43 g/L of OA in a 3-liter fermentation tank. (Jin et al. 2023). Under a multi-strategy approach, oleanolic acid was produced in a 100 L bioreactor at a yield of up to 4.07 g/L – this represents the highest yield ever reported in such studies (Cheng et al. 2024). The yields of other major active compounds from the *Ligustrum* genus in microorganisms are shown in Table 2. Although plant-based systems offer advantages such as photosynthesis, intrinsic enzyme systems, and subcellular compartmentalization, their industrial application is limited by prolonged growth cycles and intricate extraction procedures (Chen et al. 2024a, b; Vyas et al. 2025). In contrast, microbial systems demonstrate overwhelming industrial advantages, enabling rapid, high-yield production and potential cost-effectiveness through the application of metabolic engineering and optimization of fermentation processes (Hu et al. 2023; Song and Prather 2024). A fundamental requirement for these methodologies is the accurate elucidation of the structures of target compounds and their biosynthetic pathways, with a particular focus on the identification and characterization of essential catalytic enzymes, such as oxidosqualene cyclases, cytochrome P450s, and glycosyltransferases, along with their mechanisms. Comprehensive characterization of the key enzymes and regulatory mechanisms involved in Ligustrum triterpenoid biosynthesis is crucial for establishing the theoretical and technical groundwork necessary for the efficient and controllable heterologous production of these compounds in plant or microbial systems. In the future, metabolic engineering research should, following the identification of the appropriate pathways, focus primarily on further optimizing and scaling up microbial systems in order to meet the growing economic and sustainable supply demands for such compounds in the market.

**Table 2:** Biosynthesis of *Ligustrum*-specialized metabolites using metabolic engineering

Engineering bacteria	Operational methods	Products	Yield	Ref
*Yersinia lipolysis*	Overexpression of the key genes involved in the mevalonate pathway, the gene encoding cytochrome P450 (CYP716A12) to that encoding NADPH-P450 reductase	Oleanolic acid	129.9 mg/L	(Li et al. 2020)
*Saccharomyces cerevisiae*	Heterologous expression and optimization of CrAS, CrAO, and AtCPR1, and regulation of ERG1 and NADPH regeneration system	Oleanolic acid	433.9 mg/L	(Jin et al. 2023)
		Ursolic acid	1132.9 mg/L	
*Saccharomyces cerevisiae*	Balanced co-expression of CYP716A12 and ATR1, establishing a high CYP716A12-to-ATR1 ratio, boosted oleanolic acid production, together with overexpression of genes enhancing acetyl-CoA supply and NADPH regeneration	Oleanolic acid	680.8 mg/L	(Duan et al. 2024)
*Saccharomyces cerevisiae*	By deleting the galactose-metabolic genes GAL80 and GAL1 to reprogram the cellular galactose regulatory network and overexpressing 3-hydroxy-3-methylglutaryl-CoA reductase, squalene synthase, and 2,3-oxidosqualene synthase, combined with fermentation optimization	Oleanolic acid	606.9 mg/L	(Zhao et al. 2018)
*Saccharomyces cerevisiae*	HMGCR and β-lanosterol synthase were overexpressed, squalene monooxygenases optimized, ERG repressors deleted, precursors expanded, pathway rewired, diploid constructed, and the strain scaled to pilot fermentation	Oleanolic acid	4.07 g/L in a 100 L bioreactor	(Cheng et al. 2024)
*Saccharomyces cerevisiae*	A ursolic-acid module was built by linking CrMAS, CrOAS and CPR, tuning CrMAS dosage, tagging competing enzymes, deleting SSM4 to stabilize ERG1, and boosting acetyl-CoA via the Pkl–ACS route	Ursolic acid	1083 mg/L	(Zhu et al. 2025)
*Yarrowia lipolytica*	YliARO10 and YliPAR4 were retrofitted with EblHpaBC for hydroxytyrosol; mhpB was characterized then deleted, and production was boosted by precursor enrichment, pathway pruning, and temperature induction	Tyrosol	6.18 g/L in 5 L bioreactor	(Chen et al. 2025)
		Hydroxytyrosol	4.97 g/L in 5 L bioreactor	
*Escherichia coli*	Tyrosol de-novo synthesis was established by introducing ARO10/ADH6; metabolic engineering and laboratory evolution delivered a high-titer (3.3 g L⁻¹) and tolerant strain. Subsequent semi-rational tailoring of AtUGT85A1 (A21G mutation), coupled with coordinated overexpression of PGM and GalU, boosted hydrochloride glycoside de-novo production by 31.2%.	Salidroside	16.8 g/L in 5 L bioreactor	(Zeng et al. 2024)

### The pharmacological activities of the main bioactive components of ***Ligustrum***

The genus *Ligustrum* is distinguished by an extensive array of secondary metabolites, which impart a wide range of pharmacological activities, including antioxidant, anti-inflammatory, antibacterial, antitumor, antiviral, anti-osteoporotic, hypoglycemic, hypolipidemic, immunomodulatory, and hepatoprotective effects (Cao et al. 2023; Chen et al. 2024a, b; Gao et al. 2013; Yang et al. 2025).

### Anti-osteoporotic activity

**Fig. 3:**
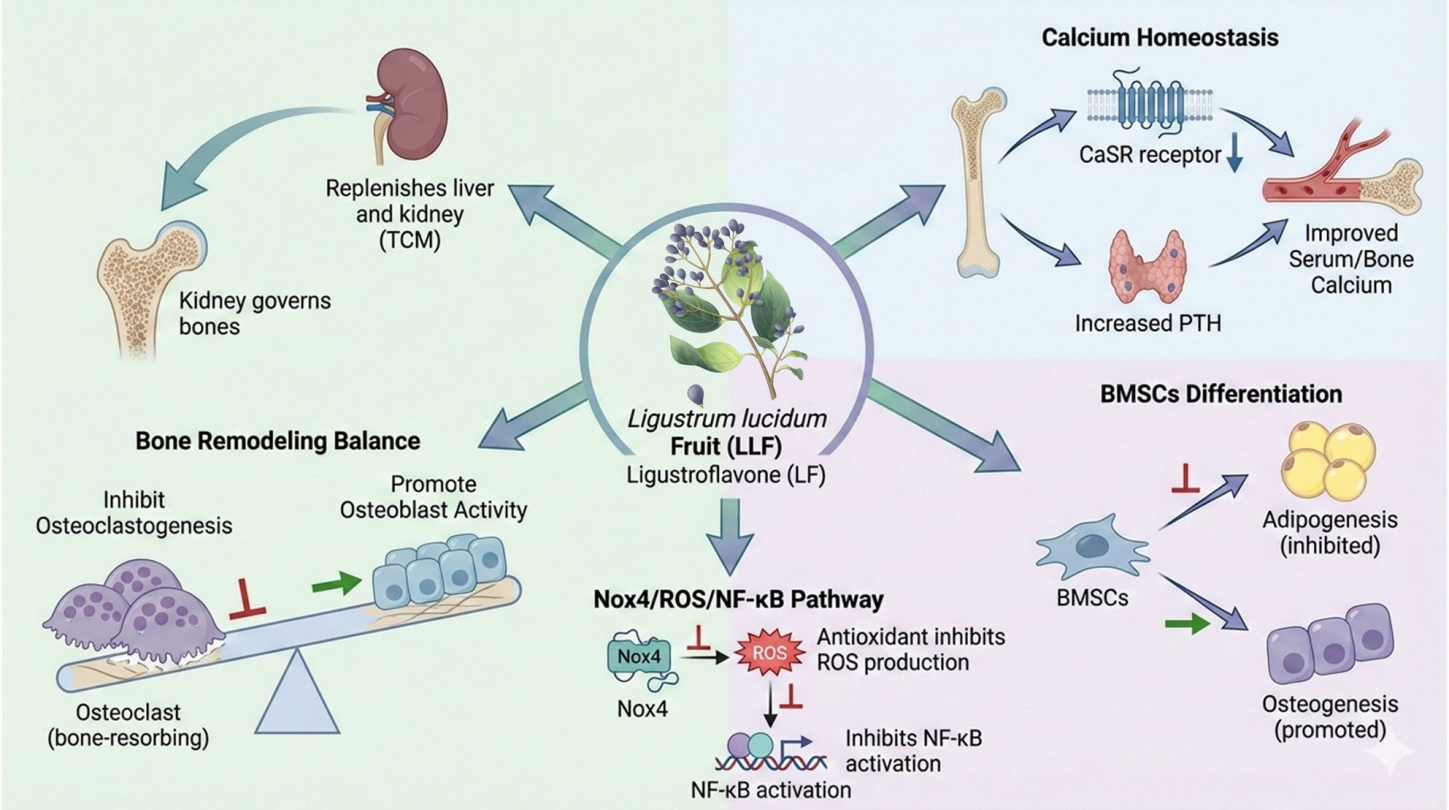
Overview of anti-osteoporosis mechanism of LLF

Osteoporosis (OP) has been identified by the World Health Organization (WHO) as one of the three principal diseases posing a significant threat to the health of middle-aged and elderly populations (Chen et al. 2018). LLF, a traditional Chinese medicinal herb known for its role in replenishing the liver and kidneys, has been extensively utilized in the prevention and treatment of osteoporosis, aligning with the TCM theory that the “kidney governs bones.” It is particularly effective in addressing postmenopausal osteoporosis (PMOP) (Qin et al. 2023). Recent pharmacological studies indicate that LLF exerts anti-osteoporotic effects through multi-target and multi-pathway mechanisms, including the modulation of bone metabolism, regulation of osteoblast and osteoclast function, and improvement of trabecular microarchitecture (Chen et al. 2024a, b; Jiang et al. 2025; Qin et al. 2023). Feng et al. demonstrated that ligustroflavone (LF), a compound isolated from LLF, significantly mitigated the diabetes-associated reduction in serum and bone calcium levels in mice, enhanced urinary calcium excretion, and increased circulating parathyroid hormone (PTH) levels. Following an eight-week regimen of LF administration, diabetic mice demonstrated significant enhancements in trabecular mineral density and microstructural indices. Mechanistic investigations revealed that LF down-regulated both mRNA and protein expression of the renal calcium-sensing receptor (CaSR), indicating that LF may act as a potential CaSR antagonist. However, a comprehensive elucidation of LF’s pharmacokinetic profile, bioavailability, clinical efficacy, and safety is yet to be conducted (Feng et al. 2019). In related research, Oh et al. showed that an extract from *L. japonicum* fruit (LJE) could attenuate bone loss by inhibiting adipogenesis and promoting the osteogenic differentiation of bone marrow mesenchymal stem cells (BMSCs) (Oh et al. 2019). Furthermore, Xu et al., utilizing a receptor activator of nuclear factor-κB ligand (RANKL)-induced osteoclast differentiation model in murine macrophage cells (RAW264.7), demonstrated that an ethanol extract of LLF significantly reduced osteoclast formation and inhibited their bone-resorbing activity, without notably affecting the apoptosis of mature osteoclasts (Xu et al. 2016). Additionally, the study conducted by Wang et al. demonstrated that an aqueous extract of LLF mitigated osteoporosis in ovariectomized (OVX) rats through various mechanisms. The findings indicated that LLF, functioning as a natural antioxidant, exerted anti-osteoporotic effects by modulating the Nox4/ROS/NF-κB signaling pathway (Wang et al. 2017) (Fig. 3).

While existing studies confirm the anti-osteoporotic potential of LLF and its components across models, these findings remain fragmented and lack systematic comparison. The use of different materials—aqueous extract of LLF, LJF, or single compounds such as LF—implies varying chemical bases and mechanisms of action, complicating direct comparisons. Moreover, reported effects—including CaSR antagonism, Nox4/ROS/NF-κB suppression, and promotion of BMSC osteogenic differentiation—have not been integrated into a coherent signaling network, leaving their interrelationships unclear. Notably, most work has focused on descriptive phenomenology and preliminary mechanisms, leaving critical gaps in the identification of key active constituents, their in vivo metabolism, target engagement, and long-term safety. Future studies should prioritize the systematic isolation, identification, and activity comparison of major chemical classes in LLF, alongside deeper investigation of crosstalk and nodal points within the implicated signaling pathways.

### Anti-tumor activity

**Fig. 4:**
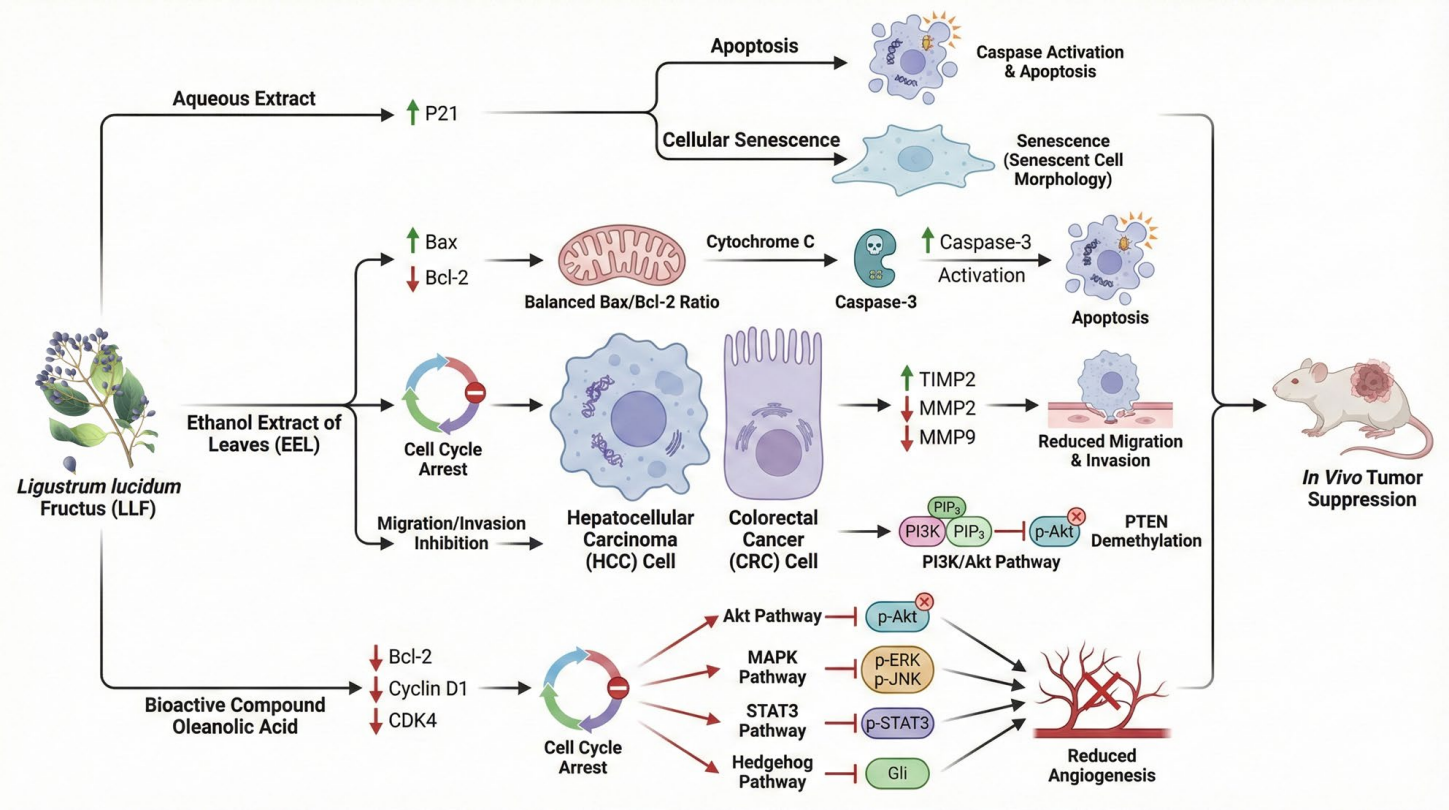
Overview of anti-tumor effects of LLF and its derivatives

LLF, recognized as a superior tonic herb in TCM, has been utilized for thousands of years and demonstrates inhibitory effects against various tumors (Lu et al. 2023). In a study by Hu et al., the effects of an aqueous extract of LLF on liver cancer were examined, revealing that it significantly induces apoptosis and cellular senescence in Bel-7402 hepatoma cells through the upregulation of P21 (Hu et al. 2014). Research conducted by Guoyan Tian et al. showed that the ethanol extract of *L. lucidum* leaves (EEL) exhibits cytotoxic effects on both Bel-7402 and Hh-7 cells by modulating the expression of Bcl-2-associated X protein (Bax), B-cell lymphoma 2 (Bcl-2), cytochrome C, and caspase-3 activity. This modulation promotes apoptosis, cell cycle arrest, and inhibits cell migration and invasion. Furthermore, EEL was found to upregulate the tissue inhibitor of metalloproteinases 2 (TIMP2) while downregulating matrix metalloproteinase 2 (MMP2) and MMP9, which further suppresses the phosphorylation of the PI3K/Akt pathway. Methylation-specific PCR analysis indicated that EEL induces PTEN demethylation, suggesting a potential link between PI3K/Akt inactivation and PTEN DNA demethylation. Subsequent in vivo investigations have corroborated the tumor-suppressive effects of the extract in hepatocellular carcinoma (Tian et al. 2019). Furthermore, oleanolic acid, a bioactive compound derived from LLF, has been shown to downregulate the expression of Bcl-2, Cyclin D1, and CDK4. It also inhibits the activation of Akt and MAPK signaling pathways and modulates various intracellular targets, thereby exerting anti-colorectal cancer effects. Additionally, oleanolic acid suppresses the signal transducer and activator of transcription 3 (STAT3) and Hedgehog signaling pathways, leading to the inhibition of angiogenesis in colorectal cancer both in vitro and in vivo  (Temby et al. 2023) (Fig. 4).

Although current studies have preliminarily revealed the anti-tumor potential of extracts from different parts of LLF, the evidence remains fragmented and lacks integration. The regulatory mechanisms involve multiple pathways such as P21/P53 and PI3K/Akt; however, the crosstalk, hierarchical relationships, and potential common upstream regulatory nodes among these pathways under LLF intervention have not yet been elucidated. Moreover, existing research is heavily focused on hepatocellular and colorectal cancer models, leaving it unknown whether the effects of LLF extend to other prevalent tumors such as breast or lung cancer.

### Anti-inflammatory and analgesic activity

**Fig. 5:**
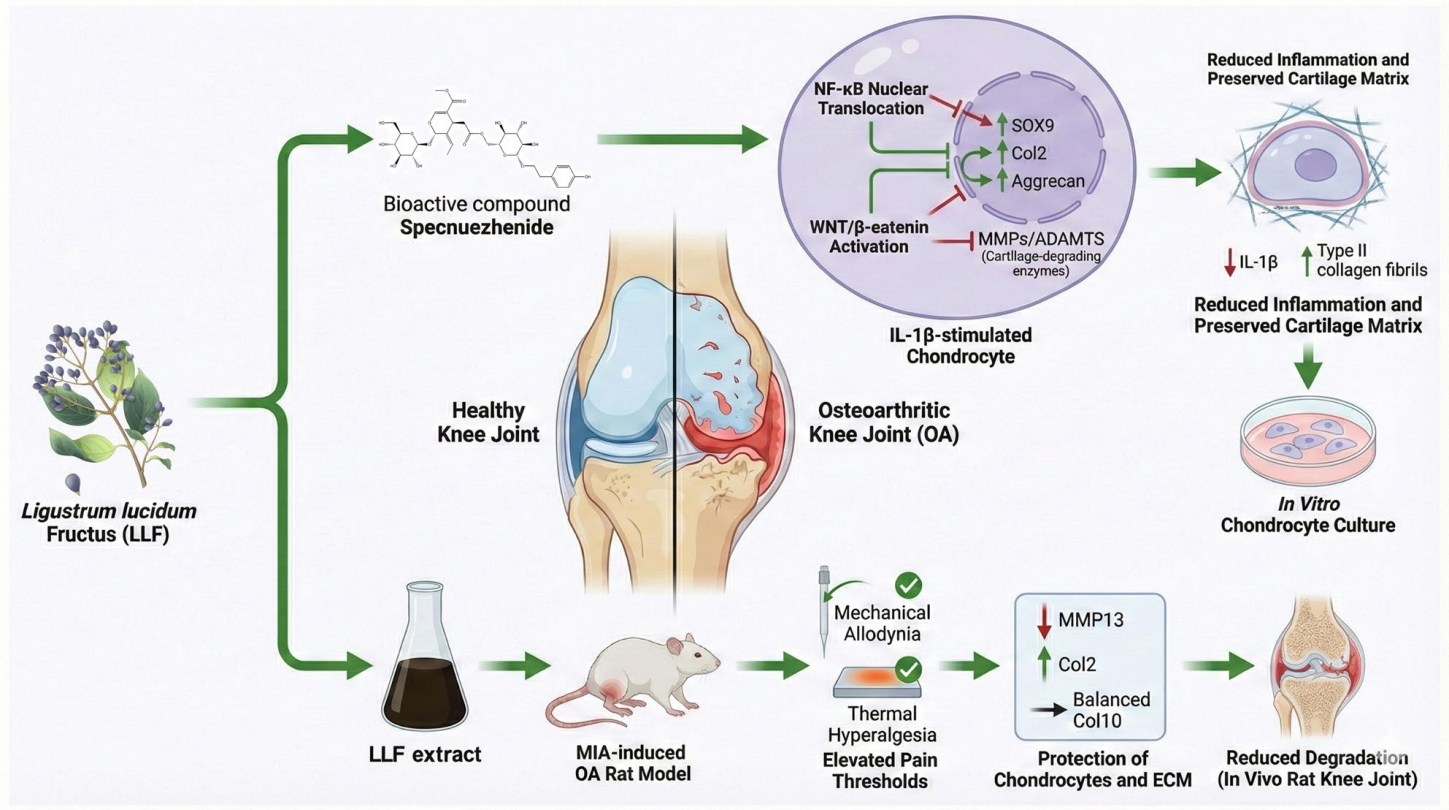
Overview of anti-osteoarthritic effects of LLF derivatives in OA

Osteoarthritis (OA), a highly prevalent degenerative joint disorder, is characterized by the degradation of cartilage, synovial inflammation, and chronic pain, with the pro-inflammatory cytokines IL-1β and TNF-α playing crucial roles in its pathogenesis. In their study, Chiyuan Ma et al. explored the anti-inflammatory effects and underlying mechanisms of specnuezhenide, a bioactive compound derived from LLF, in the context of OA through both in vitro and in vivo experiments. The results of their research indicated that specnuezhenide mitigates IL-1β-induced chondrocyte inflammation by (1) downregulating the expression of cartilage-degrading enzymes, (2) inhibiting the activation of the NF-κB and WNT/β-catenin signaling pathways, and (3) upregulating the expression of chondrocyte-specific genes, thereby exhibiting therapeutic potential against OA (Ma et al. 2018). Furthermore, LLF demonstrated analgesic properties in a rat model of OA induced by monosodium iodoacetate (MIA). Following the intra-articular administration of MIA at a concentration of 100 µg/mL, LLF significantly mitigated joint pain hypersensitivity by elevating the thresholds for mechanical allodynia, thermal hyperalgesia, and spontaneous movement. On a mechanistic level, LLF counteracted OA-related cartilage degradation through two primary actions: (1) safeguarding chondrocytes and the extracellular matrix, and (2) modulating the expression of critical cartilage remodeling proteins, including matrix metalloproteinase 13 (MMP13), collagen type II (Col2), and collagen type X (Col10) (Yan et al. 2018) (Fig. 5).

Although the aforementioned studies reveal the positive effects of specnuezhenide and LLF extracts in OA models, the research remains highly focused on a single compound, and existing in vivo models primarily rely on MIA-induced acute injury. It is questionable whether this model adequately simulates the complex, chronic, and multifactorial (e.g., aging, mechanical load) pathological progression of human OA, thus limiting the generalizability of the findings. Meanwhile, studies have predominantly focused on cartilage protection, while exploration of their impact on another core pathological aspect of OA—synovial inflammation and fibrosis—remains insufficient. Future research should address the aforementioned limitations by employing multi-omics technologies and gene-editing tools to integrate the identified signaling pathways, construct their regulatory networks, and validate key nodes. Particular emphasis should be placed on investigating joint-targeted delivery and the pharmacokinetics of active constituents to provide a foundation for clinical translation.

### Hypoglycemic and lipid-regulating activity

**Fig. 6:**
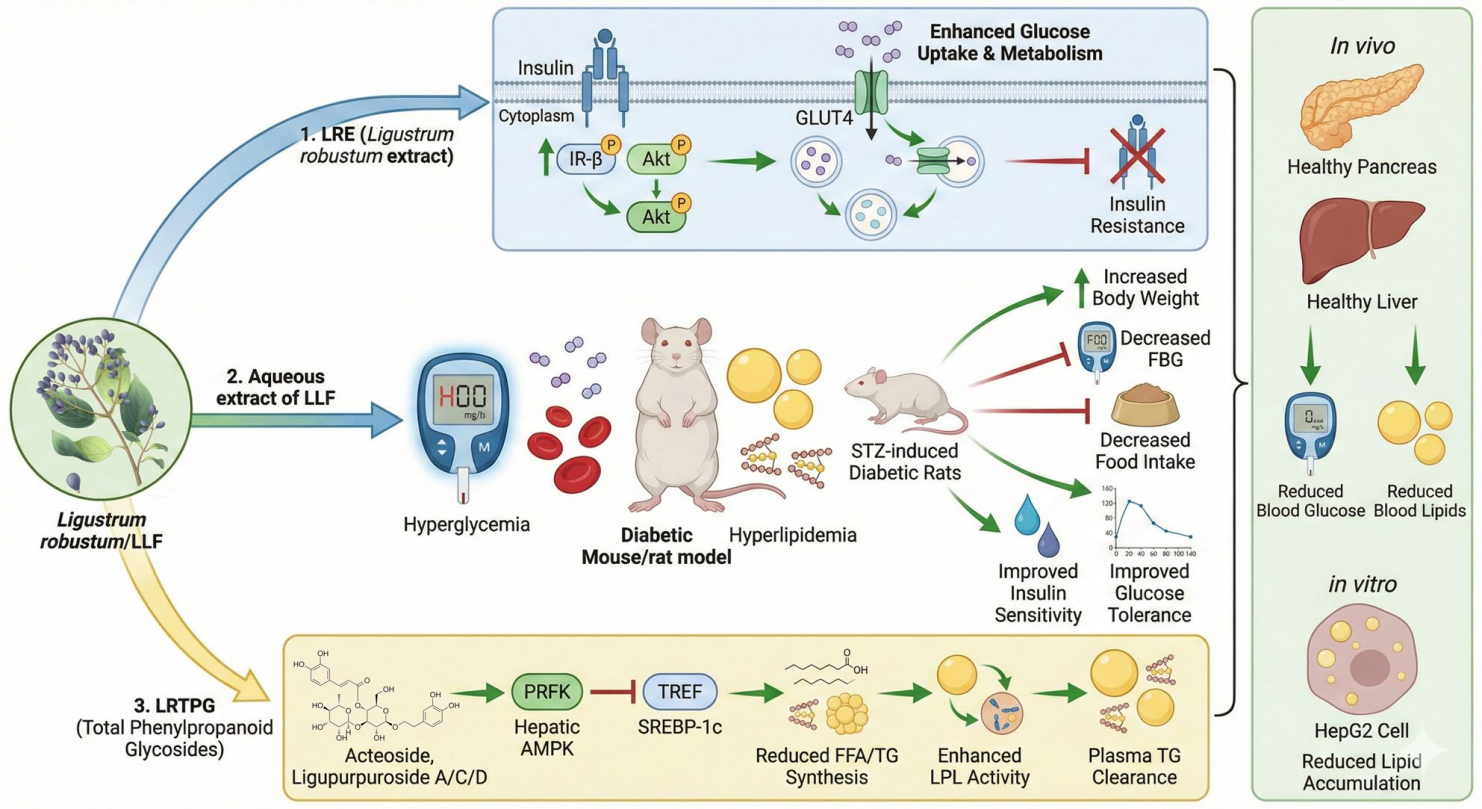
Overview of hypoglycemic and hypolipidemic effects of LLF in diabetes

Diabetes mellitus (DM), a metabolic disorder marked by persistent hyperglycemia, represents a substantial global health challenge. In a study conducted by Cao Fang et al., the hypoglycemic mechanisms of *Ligustrum robustum* extract (LRE) were examined using an alloxan-induced diabetic mouse model. The findings revealed that LRE significantly decreased blood glucose levels in mice with type 2 diabetes mellitus (T2DM). The flavonoids contained in LRE were found to upregulate and activate the insulin receptor (IR-β) and protein kinase B (PKB/Akt) signaling pathways, which facilitated the translocation of glucose transporter 4 (GLUT4). This process resulted in enhanced glucose and lipid metabolism and a reduction in insulin resistance in T2DM mice. Furthermore, clinical observations corroborated that the oral administration of Ligustrum robustum leaves effectively reduces blood glucose levels (Cao et al. 2016). In a similar vein, Lv et al. demonstrated that an aqueous extract of LLF mitigated symptoms in diabetic rats induced by streptozotocin (STZ). Administration of the extract resulted in a significant reduction in food intake (FI) and fasting blood glucose (FBG) levels, alongside an increase in body weight (BW). Furthermore, enhancements in insulin sensitivity and glucose tolerance were noted (Lv et al. 2018). In a separate investigation, Yang et al. examined the lipid-lowering properties of total phenylpropanoid glycosides (LRTPG) derived from *Ligustrum robustum*. LRTPG significantly decreased both plasma and hepatic lipid concentrations, with a pronounced effect on triglycerides (TGs). At a concentration of 50 µmol/L, the principal bioactive components of LRTPG—namely, acteoside (verbascoside) and ligupurpuroside A, C, and D—effectively inhibited oleic acid-induced lipid accumulation in human hepatoma HepG2 cells. Mechanistically, LRTPG mediates its hypolipidemic effects through two primary pathways: (1) activation of the hepatic AMPK/SREBP-1c pathway, which suppresses the synthesis of free fatty acids (FFA) and TG, and (2) enhancement of lipoprotein lipase (LPL) activity in hamsters, thereby facilitating plasma TG clearance. These findings indicate that LRTPG holds significant potential as a therapeutic agent for the treatment of hyperlipidemia, particularly in cases of dyslipidemia associated with diabetes and obesity (Yang et al. 2018) (Fig. 6).

Although current studies have confirmed the hypoglycemic, insulin resistance-improving, and lipid-lowering activities of various *Ligustrum* extracts in multiple diabetic animal models, the evidence remains notably fragmented and heterogeneous. Future research should employ standardized extracts or well-defined compound combinations for parallel comparative studies in unified diabetic models to identify the principal active substances. Utilizing metabolomics, transcriptomics, and network pharmacology approaches can help integrate the currently dispersed signaling pathways, construct their regulatory networks, and validate key cross-talk nodes. Furthermore, expanding the research scope to systematically evaluate the protective effects and underlying mechanisms of *Ligustrum* extracts against major diabetic complications is essential.

### Other pharmacological activities

In addition to the previously discussed effects, *Ligustrum* species exhibit several other significant pharmacological activities. Research has demonstrated that an aqueous extract of LLF markedly decreases serum alanine aminotransferase (ALT) levels in mice and downregulates the expression of genes associated with liver inflammation, such as EGF-1 (Su et al. 2017). Seo et al. explored the antioxidant and mitochondrial-protective properties of LLF extract in both in vitro and in vivo settings. Their results indicated that the extract alleviated oxidative stress-induced hepatocellular damage by modulating the AMPK signaling pathway (Seo et al. 2017). Moreover, Ye et al. investigated the neuroprotective potential of LLF in a yeast model of Parkinson’s disease (PD). The treatment with the extract improved cellular viability, diminished reactive oxygen species (ROS) accumulation, and activated glutathione peroxidase (GPx), indicating its potential therapeutic application for neurodegenerative disorders like PD (Ye et al. 2016). Furthermore, phenol glycosides (PG) extracted from LLF have been demonstrated to mitigate lipopolysaccharide (LPS)-induced depressive-like behaviors in murine models. Mechanistically, pretreatment with PG inhibited neuroinflammation in the hypothalamus by (1) suppressing microglial activation and the production of pro-inflammatory cytokines through the TLR4 pathway, the CaSR, and the renin-angiotensin system (RAS) cascade, and (2) modulating vitamin D metabolism. These findings underscore the potential of PG as a novel antidepressant agent (Feng et al. 2020).

Although the aforementioned studies have preliminarily revealed the multifaceted pharmacological activities of LLF, such as hepatoprotective, antioxidant, neuroprotective, and antidepressant effects, the current knowledge system remains notably fragmented and isolated. Most research has only progressed to describing single pathways or phenotypes, lacking in-depth exploration of cross-pathway interactions and upstream initiating signals. Future pharmacological studies on LLF should be more comprehensive and in-depth to lay a foundation for the subsequent development of multi-target, multifunctional botanical drugs or health products based on this plant.

## Limitations and translational challenges of pharmacological studies

While extensive preclinical studies have revealed the broad therapeutic potential of *Ligustrum* extracts and their monomeric compounds, significant challenges remain in successfully translating them into clinical applications. Firstly, most evidence is based on crude extracts or total glycoside fractions, where active constituents are ambiguous, making it difficult to identify the exact pharmacologically effective components. Secondly, existing research primarily involves in vitro cell experiments or animal models, and the effective doses (such as in vitro IC50 values or in vivo mg/kg doses) remain unclear regarding their physiological accessibility in humans, oral bioavailability, and systemic pharmacokinetic characteristics. Additionally, there is a lack of long-term toxicological data on key active ingredients (e.g., specnuezhenide, luteolin, oleanolic acid) and well-designed clinical trials based on pure compounds. Therefore, future research should focus on: (1) identifying key monomeric compounds from active extracts; (2) conducting systematic ADMET (absorption, distribution, metabolism, excretion, toxicity) evaluations of lead compounds; (3) developing formulation studies to improve the bioavailability of poorly water-soluble components; (4) ultimately advancing to clinical validation stages. Only through such translational research can the traditional medicinal value of *Ligustrum* be transformed into modern evidence-based drugs.

## Conclusion and future perspectives

The genus *Ligustrum*, recognized for its medicinal and edible value, demonstrates significant diversity in bioactive constituents across its species. This review systematically compiles the primary secondary metabolites present in *Ligustrum* plants, highlighting key compounds such as terpenoids, flavonoid derivatives, and phenylethanoid glycosides. These constituents collectively underpin the pharmacological foundation for the genus’s multi-target and multi-pathway bioactivities, which include, but are not limited to, anti-osteoporosis, antioxidant, hepatoprotective, anti-inflammatory, and antitumor effects. The demonstrated bioactivities, coupled with the successful heterologous production of key compounds like salidroside and oleanolic acid in microbial systems, underscore a clear translational pathway from botanical research to industrial manufacture.

Despite its extensive history in traditional medicinal systems and its confirmed therapeutic potential through contemporary pharmacological research, several critical scientific challenges persist in the translation of *Ligustrum* into modern pharmaceuticals and the functional food industry. From the standpoint of sustainable resource management, the escalating medicinal significance of *Ligustrum* necessitates the urgent development of scientifically grounded cultivation and domestication strategies to conserve wild populations and cultivate superior germplasm. Importantly, the chemical compositions of the stem and leaf tissues within this genus exhibit substantial similarity to those of the fruits, offering a theoretical basis for extending the range of exploitable plant organs and enhancing resource utilization efficiency. Nonetheless, the intrinsic bitterness of these tissues poses a considerable barrier to their application in the food industry. Consequently, future research should prioritize the development of innovative purification or biotransformation techniques to improve palatability, thereby creating palatable, standardized extracts suitable for functional food and nutraceutical product development.

At the molecular level, the integration of multi-omics approaches is essential to elucidate unresolved biosynthetic pathways. The primary industrial application of this foundational knowledge lies in the engineering of scalable microbial or plant cell factories. Although the biosynthesis of specific triterpenoids, such as oleanolic acid and ursolic acid, and phenylethanoid glycosides, such as salidroside, has been elucidated and effectively applied in metabolic engineering, the genetic foundations and regulatory mechanisms underlying critical reactions, including flavonoid C-glycosylation, phenylethanoid hydroxylation, and triterpenoid post-modification, remain insufficiently understood. The integration of multi-omics approaches—encompassing genomics, transcriptomics, metabolomics, and epigenomics—to develop a comprehensive “gene-enzyme-metabolite-phenotype” association network is essential. This integration would not only create a valuable repository for synthetic biology and metabolic engineering but also establish a robust multi-omics research platform for *Ligustrum*, addressing current research bottlenecks. Such advancements have the potential to yield novel insights into the regulation of secondary metabolism and facilitate breakthroughs in drug development and the promotion of human health. Future metabolic engineering efforts must not only aim for high titer, rate, and yield, but also address cost-effectiveness at scale, substrate affordability, and the simplification of downstream processing to meet industrial benchmarks.

Consequently, a coordinated future research agenda should span advanced scientific exploration, engineered production solutions, and practical product development. This entails deepening multi-omics studies to resolve fundamental biosynthetic pathways, concurrently applying this knowledge to engineer robust microbial or plant cell factories that are optimized for industrial scalability and cost-effectiveness, and parallelly advancing the development of standardized extracts and formulations through rigorous studies on stability, safety, and regulatory compliance. This tripartite strategy will effectively bridge the gap between traditional use and modern validation, transforming *Ligustrum* from a regionally utilized herb into a globally recognized, high-value resource. Ultimately, the convergence of rigorous science, efficient manufacturing, and robust regulatory strategy will unlock the full commercial and therapeutic potential of *Ligustrum*, offering innovative, evidence-based solutions for human health and sustainable development.

## Data Availability

All data used in this manuscript are publicly available.

## Abbreviations

HMG-CoA: Hydroxymethylglutaryl-CoA
MVA: Mevalonate
MVA-5P: Mevalonate-5-phosphate
MVA-5PP: Mevalonate-5-pyrophosphate
IPP: Isopentenyl diphosphate
D-G3P: D-Glyceraldehyde 3-phosphate
DXP: 1-Deoxy-D-xylulose 5-phosphate
MEP: 2-C-Methyl-D-erythritol 4-phosphate
CM: 4-(Cytidine 5-diphospho)-2-C-methyl-D-erythritol
PCM: 2-Phospho-4-(cytidine 5-diphospho)-2-C-methyl-D-erythritol
MECDP: 2-C-Methyl-D-erythritol 2,4-cyclodiphosphate
HDMAPP: 1-Hydroxy-2-methyl-2-butenyl 4-diphosphate
DMAPP: Dimethylallyl diphosphate
AACT: Acetyl-CoA C-acetyltransferase
HMGCS: Hydroxymethylglutaryl-CoA synthase
HMGCR: Hydroxymethylglutaryl-CoA reductase
MVK: Mevalonate kinase
PMK: Phosphomevalonate kinase
MVD: Diphosphomevalonate decarboxylase
FPPS: Farnesyl pyrophosphate
FPPS: Farnesyl pyrophosphate synthase
SQS: Squalene synthase
SQE: Squalene epoxidase
β-AS: β-amyrin synthase
α-AS: α-amyrin synthase
DXS: 1-Deoxy-D-xylulose-5-phosphate synthase
DXR: 1-Deoxy-D-xylulose-5-phosphate reductase
MCT: 2-C-Methyl-D-erythritol 4-phosphate cytidylyltransferase
CMK: 4-Diphosphocytidyl-2-C-methyl-D-erythritol kinase
MDS: 2-C-Methyl-D-erythritol 2,4-cyclodiphosphate synthase
HDS: (E)-4-Hydroxy-3-methylbut-2-enyl-diphosphate synthase
HDR: 4-Hydroxy-3-methylbut-2-en-1-yl diphosphate reductase
IDI: Isopentenyl diphosphate isomerase
GPP: Geranyl diphosphate
GES: Geraniol synthase
G10H: Geraniol 10-hydroxylase
10GHO: 10-Hydroxygeraniol oxidoreductase
ISY: Iridoid synthase
IO: Iridoid oxidase
7DLGT: 7-Deoxyloganetic acid glucosyltransferase
7eDLH: Epi-deoxyloganic acid 7-hydroxylase
eLAMT: Epi-loganic acid O-methyltransferase
SXS: Secoxyloganin synthase
OMES: Oleoside methyl ester synthase
UDP-GT: UDP-glycosyltransferase
4-HPAA: 4-Hydroxyphenylacetaldehyde
TyDC: Tyrosine/dopa decarboxylase
MAO: Monoamino oxidase
AAS: Aromatic aldehyde synthase
PAR: Phenylacetaldehyde reductase
TCM: Traditional Chinese medicine
LLF: Ligustri Lucidi Fructus
GPP: Geranyl pyrophosphate
OME: Oleoside-11-methyl ester
T8GT: UDP-glucose 8-O-glucosyltransferase
4-HPAAS: 4-Hydroxyphenylacetaldehyde synthase
AAS: Aromatic aldehyde synthase
AAD: Aaromatic amino acid decarboxylase
OP: Osteoporosis
WHO: World Health Organization
PMOP: Postmenopausal osteoporosis
LF: Ligustroflavone
PTH: Parathyroid hormone
CaSR: Calcium-sensing receptor
LJE: L. japonicum fruit
BMSC: Bone marrow mesenchymal stem cell
RANKL: Factor-κB ligand
OVX: Ovariectomized
EEL: Ethanol extract of L. lucidum leaves
Bcl-2: B-cell lymphoma 2
Bax: Bcl-2-associated X protein
TIMP2: Tissue inhibitor of metalloproteinases 2
MMP2: Matrix metalloproteinase 2
STAT3: Signal transducer and activator of transcription 3
OA: Osteoarthritis
MIA: Monosodium iodoacetate
MMP13: Metalloproteinase 13
Col2: collagen type II
Col10: collagen type X
DM: Diabetes mellitus
LRE: Ligustrum robustum extract
T2DM: Type 2 diabetes mellitus
IR-β: Insulin receptor
GLUT4: Glucose transporter 4
STZ: Streptozotocin
FI: Food intake
FBG: Fasting blood glucose
BW: Body weight
LRTPG: Phenylpropanoid glycosides
TG: Triglyceride
FFA: Free fatty acids
LPL: Lipoprotein lipase
ALT: Alanine aminotransferase
PD: Parkinson’s disease
GPx: Glutathione peroxidase
PG: Phenol glycosides
LPS: Lipopolysaccharide
RAS: Renin-angiotensin system
